# Supplementary material for: Phylogenetic Relationships and Evolution of the Genus Eganvirus (186-Type) Yersinia pestis Bacteriophages
Source: Viruses. 2024 May 8;16(5):748. doi: 10.3390/v16050748 (PMC11126057; doi:10.3390/v16050748)
Supplement: Supplementary file 1 [file viruses-16-00748-s001.zip › Supplementary Table S2-202403-09-liwei.pdf]

Supplementary S2. ***Eganvirus* phages and other P2-like phages in NCBI used in phylogenetic analysis.**

No.	Name	Known Genus	Host strain/source	Country	GC (%)	Sequence Size (bp)	Accession No.	Genes Existence				Ref.
								int	c/cI	cox/ alp	cII	
**Bacteriophages 1: genus *Eganvirus***												
1	186	Eganvirus	*Escherichia coli*	USA	53.1	30624	NC_001317	√	√	√	√	1
2	EtG	Eganvirus	*Erwinia tracheiphila* Et-B, Cucumis sativus	Pakistan	54.1	30413	NC_047833	√	√	√	√	8,18
3	PsP3	Eganvirus	*Salmonella enterica* subsp. *enterica*	Potsdam	52.8	30636	NC_005340	√	√	√	√	1,13
4	SEN1	Eganvirus	*Salmonella enterica* subsp. *salamae* 10/99 II	Czech Republic	53.0	29733	KT630644.2	√	√	√*	√	9
5	SW9	Eganvirus	*Salmonella* sp.	Canada	53.0	31123	MK972711	√	√	√*	√	14
6	BIS20	Eganvirus	*Salmonella enterica* subsp. *enterica* serovar *Typhimurium* SE-BS17	NA	53.2	29512	NC_070911	√	√	√	√	15
7	SI22	Eganvirus	*Salmonella* sp.	Canada	53.0	30920	MK972710	√	√	√*	√	—
8	vB_EcoM-613 R3	NA	*Escherichia coli*	United Kingdom	51.2	44992	ON470592	√	√	√*	√	—
9	vB_Sal_PHB48	NA	*Salmonella enterica* subsp. *enterica* serovar *Enteritidis* CICC 21482	China	52.4	29575	ON782130	√	√	√	√	16
**Bacteriophages 2: 186-type phages or P2-like phages**												
1	ENT90	Entnonagintavirus	*Erwinia amylovora*	China	55.8	29564	NC_019932	√	√	√	√	17,18
2	Fels-2	Felsduovirus	*Salmonella typhimurium* LT2	NA	52.5	33693	NC_010463	√	√	√	√	1
3	SopE ϕ	Felsduovirus	*Salmonella typhimurium* DT204	—	51.3	35155	AY319521	√	√	√	√	1
4	3625_26581	NA	Bacteriophage sp. isolate 3625_26581	Japan	54.7	27828	OP075671	√	√	√*	√	—

5	K139	Longwoodvirus	*Vibrio cholerae* O139	NA	48.9	33106	NC_003313	√	√	√	√	1,24
6	Kappa	Longwoodvirus	*Vibrio cholerae O1 biovar eltor*	NA	48.8	33507	NC_010275					1,23
7	ϕO18P	Bielevirus	*Aeromonas media*	NA	61.7	33985	NC_009542					1,24
8	HP1	Hpunavirus	*Haemophilus influenzae*	NA	40.0	32355	NC_001697					1,19,20,21
9	HP2	Hpunavirus	*Haemophilus influenzae*	NA	39.9	31508	NC_003315					1,21
10	F108	Irtavirus	*Pasteurella multocida*	NA	42.1	30505	NC_008193			—		1,22
**Bacteriophages 3: genus *Peduovirus***												
1	P2	Peduovirus	*Escherichia coli*	NA	50.2	33593	NC_001895	√	√	√	√	1,2,4,12,13
2	P2_Hy_dis	Peduovirus	Recombinant between P2 and a defective P2-like prophage in an *Escherichia coli* B strain	NA	—	—	AJ306907-c&cox	√	√	√	√	2,7,10,11
3	ϕD124	Peduovirus	*Escherichia coli* C, Clinical isolate	Los Angeles, Calif	—	—	AM159060-c&cox, AJ298558-ONML	√	√	√	√	1,2
4	Wϕ	Peduovirus	*Escherichia coli* W str. ATCC9637	NA	51.7	32684	NC_005056	√	√	√	√	1,2
5	L-413C	Peduovirus	*Yersinia pestis*	NA	52.1	30728	NC_004745	√	√	√	√	1,2,4
6	ϕD160	Peduovirus	*Escherichia coli* C, Clinical isolate	Los Angeles, Calif	—	—	AM159075-c&cox, AJ298560-ONML	√	√	√	√	1,2
7	ϕD145	Peduovirus	*Escherichia coli* C, Clinical isolate	Los Angeles, Calif	—	—	AM159077-c&cox, AM158280-int, AJ298559-ONML	√	√	√	√	1,2
8	P2-EC4	Peduovirus	*Escherichia coli str.* ECOR4	NA	—	—	AM159080-c&cox	√	√	√	√	2
9	GMG73	Peduovirus	*Yersinia pestis*	China	51.5	33804	OP880301	√	√	√	√	4
10	GMS190	Peduovirus	*Yersinia pestis*	China	53.0	29462	OP880302	√	√	√	√	4
11	HQ103	Peduovirus	Yersinia pestis EV76-CN	China	51.7	31962	NC_070908	√	√	√	√	4,6

12	Vb_YpM_22	Peduovirus	*Yersinia pestis* EV76-CN	China	51.5	31809	NC_055844	√	√	√	√	3,4
13	Vb_YpM_46	Peduovirus	*Yersinia pestis* EV76-CN	China	51.7	32336	NC_055845	√	√	√	√	3,4
14	Vb_YpM_50	Peduovirus	*Yersinia pestis* EV76-CN	China	52.1	31096	NC_055846	√	√	√	√	3,4
15	vB_YenM_42.18	Peduovirus	*Yersinia enterocolitica*	Germany	46.4	36481	NC_070913					5
**Bacteriophages 4: Prophages**												
	Name	Host strain/source		Location in host genome	GC (%)	Sequence Size (bp)	Sequence accession No.					Ref.
1	Φ ECA29	*Erwinia carotovora* subsp. *atroseptica* SCRI1043		2935258-2966778	50.9	31521	NC_004547					1
2	φ ECO1	*Escherichia coli* HS		916812-948276	52.0	31465	NC_009800	√	√	√	√	1
3	φ ECO2	*Escherichia coli* CATCC 8739		3023202-3058101	50.5	34900	NC_010468	√	√	√	√	1
4	φ ECO3	*Escherichia coli* CFT073		908870-942351	50.3	33482	NC_004431	√	√	√	√	1
5	φ ECO4	*Escherichia coli E22*		1631226-1670396	51.7	39171	NZ_AAJV00000000					1
6	φ ECO5	*Escherichia coli* UTI89		903605-951760	53.5	32767	NC_007946					1
7	φ ECO6	*Escherichia coli* 101-1		908551-947403	52.6	38853	NZ_AAMK00000000					1
8	φ ECO7	*Escherichia coli* 53638		2139828-2172764	51.9	32927	NZ_AAKB00000000					1
9	φ ESP	*Enterobacter* sp. 638		3783460-3815505	53.1	32093	NC_009436	√	√	√	√	1
10	Φ YPS	*Yersinia pseudotuberculosis* IP 32953		3688279-3717980	49.2	29702	NC_006155					1
11	Φ YE98	*Yersinia enterocolitica* subsp. *enterocolitica* 8081		981219-1012614	48.8	31396	NC_008800					1
12	φ KPN	*Klebsiella pneumoniae* subsp. *pneumoniae* MGH 78578		3803150-3835929	52.0	32827	NC_009648	√	√	√	√	1
13	φ SEN1	*Salmonella enterica* subsp. *enterica serovar* Typhi CT18		3515381-3549053	51.8	33673	NC_003198	√	√	√	√	1
14	φ SEN2	*Salmonella enterica* subsp. *enterica serovar* Typhi CT18		4473835-4507389	51.7	32767	NC_003198	√	√	√	√	1

notes："NA" denotes not applicable, "—" indicates the absence of content, "-c&cox" refers to the accession numbers for the C and Cox genes, "-ONML" specifies the accession numbers for the O, N, M, and L genes, while entries without any suffix represent the accession numbers for the complete genome sequences.

Reference.

1. Phylogenetic structure and evolution of regulatory genes and integrases of P2-like phages
2. Evolution of Immunity and Host Chromosome Integration Site of P2-Like Coliphages
3. Identification and characterization of P2-like bacteriophages of Yersinia pestis
4. A temperate P2-like bacteriophage that originated from Escherichia coli can infect Yersinia pestis
5. Host Range, Morphology and Sequence Analysis of Ten Temperate Phages Isolated from Pathogenic Yersinia enterocolitica Strains
6. Temperature-dependent carrier state mediated by H-NS promotes the long-term coexistence of Y. pestis and a phage in soil\
7. Characterization of the developmental switch region of bacteriophage P2 Hy dis
8. Complete Genome Sequence of EtG, the First Phage Sequenced from Erwinia tracheiphila
9. Phages of Salmonella enterica subsp. salamae and subsp. diarizonae: novel phages with a mosaic genome structure and activity against pathogenic S. enterica subsp. enterica isolates
10. Electron microscope heteroduplex mapping of P2 Hy dis bacteriophage DNA
11. A variant of phage P2 originating in Escherichia coli, strain B
12. Studies on lysogenesis. I. The mode of phage liberation by lysogenic Escherichia coli
13. Characterization of five novel endolysins from Gram-negative infecting bacteriophages
14. Genomic Features and Phylogenetic Analysis of Antimicrobial-Resistant Salmonella Mbandaka ST413 Strains
15. Phenotypic characterization and genome analysis of a novel Salmonella Typhimurium phage having unique tail fiber genes
16. Temperate phage influence virulence and biofilm-forming of Salmonella Typhimurium and enhance the ability to contaminate food product
17. Contributions of P2- and P22-like prophages to understanding the enormous diversity and abundance of tailed bacteriophages
18. Novel Erwinia persicina Infecting Phage Midgardsormr38 Within the Context of Temperate Erwinia Phages
19. Haemophilus influenzae HP1 Bacteriophage Encodes a Lytic Cassette with a Pinholin and a Signal-Arrest-Release Endolysin
20. The complete nucleotide sequence of bacteriophage HP1 DNA
21. Bacteriophage HP2 of Haemophilus influenzae

22. Isolation and sequencing of a temperate transducing phage for Pasteurella multocida
23. Temperature-sensitive mutants of serratia phage Kappa
24. Temperate bacteriophage PhiO18P from an Aeromonas media isolate: characterization and complete genome sequence
