# Supplementary figures and images for: Phylogenetic Relationships and Evolution of the Genus Eganvirus (186-Type) Yersinia pestis Bacteriophages
Source: Viruses. 2024 May 8;16(5):748. doi: 10.3390/v16050748 (PMC11126057; doi:10.3390/v16050748)

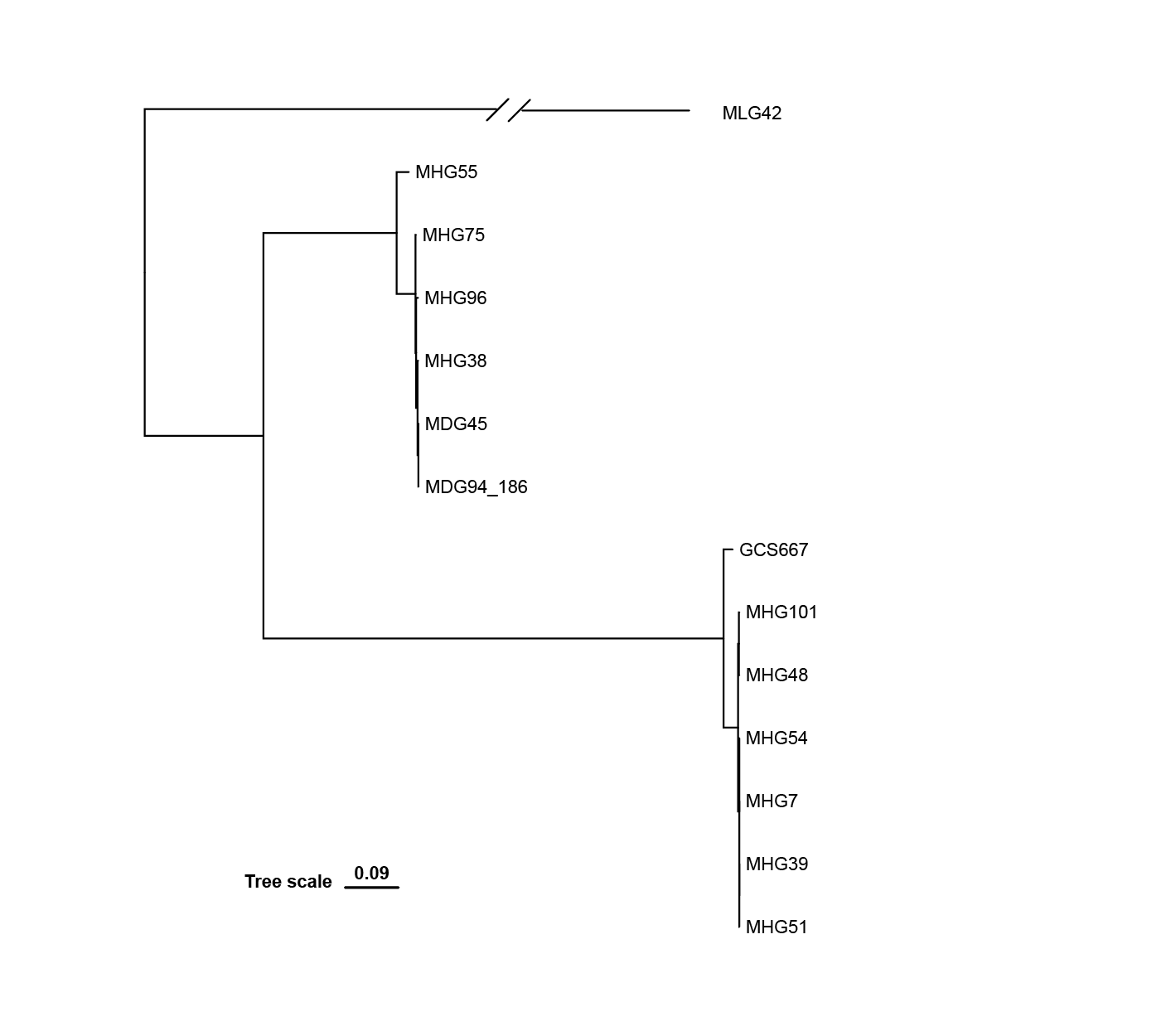

Supplement: Supplementary file 1 [file viruses-16-00748-s001.zip › Supplemental Figure S1-Detailed Phylogenetic relationship .tif]

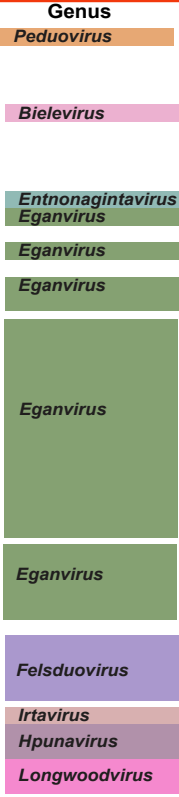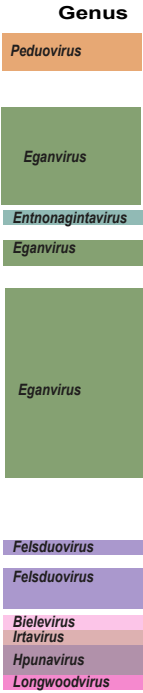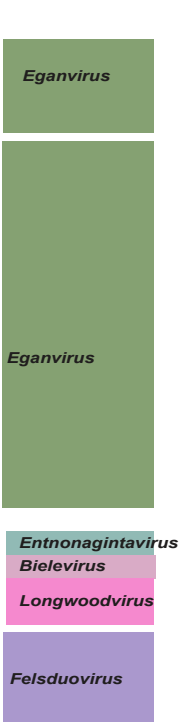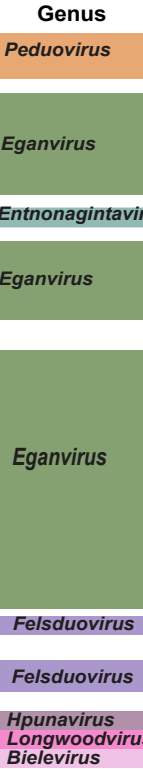

Supplement: Supplementary file 1 [file viruses-16-00748-s001.zip › Supplemental Figure S2- Phylogenetic -Regulatory region-4-19/Supplemental figure S2-2024-4-29.pdf]

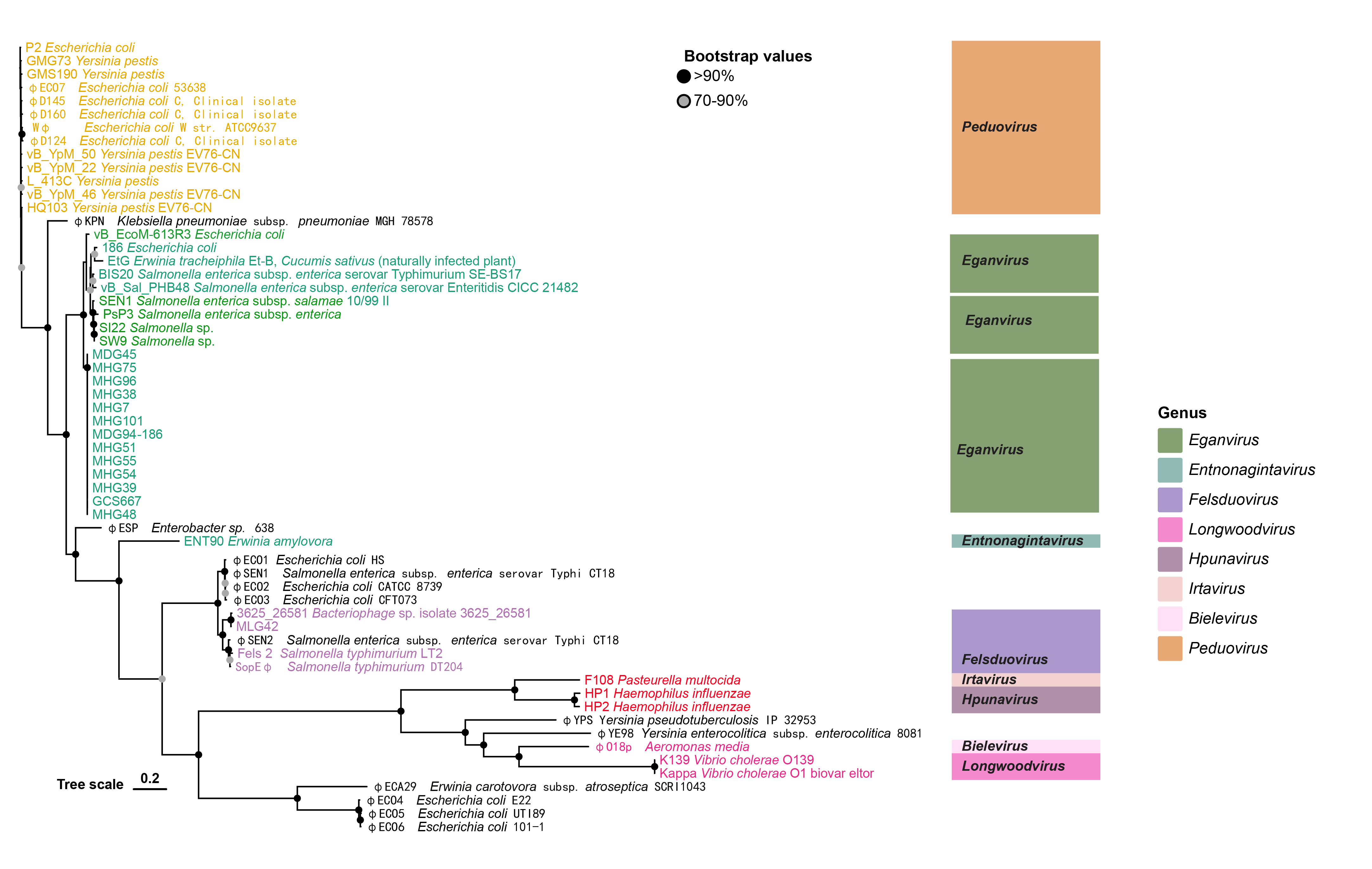

Supplement: Supplementary file 1 [file viruses-16-00748-s001.zip › Supplemental Figure S3- Phylogenetic relationship structural genes.tif]
